# Editorial Comment: The effect of shock wave lithotripsy and retrograde intrarenal surgery on health-related quality of life in 10-20 mm renal stones: a prospective randomized pilot study

**DOI:** 10.1590/S1677-5538.IBJU.2022.01.07

**Published:** 2021-12-21

**Authors:** Alexandre Danilovic

**Affiliations:** 1 Faculdade de Medicina da Universidade de São Paulo Hospital das Clínicas Departamento de Urología São Paulo SP Brasil Departamento de Urología, Hospital das Clínicas, Faculdade de Medicina da Universidade de São Paulo, São Paulo, SP, Brasil

Gokhan Atis 1, Meftun Culpan 2, Taha Ucar 1, Furkan Sendogan 3, Huseyin Ozgur Kazan 1, Asif Yildirim 1

Urolithiasis. 2021 Jun;49(3):247-253.

## COMMENT

Patients with urolithiasis have significantly lower health-related quality of life (HRQoL) and the modality of treatment may also have impact on HRQoL (1). AUA and EAU Guidelines for stone disease consider stone free rate and complication rate in the recommendation for surgical management of urinary stones (2, 3). However, there are some clinical situations which the treatment modality has similar stone free rate and complication rate. Patient related outcomes such as HRQoL are rarely studied in randomized clinical trials (RCT) of stone intervention management and more high quality studies are welcome.

Atis et al. compared the effect of retrograde intrarenal surgery (RIRS) and shockwave lithotripsy (SWL) on health-related quality of life (HRQoL) in patients with renal stones between 10 and 20 mm (4). A total of 120 patients were prospectively randomized. Only successfully treated patients (75%, 45/60 in RIRS group and 60%, 36/60 in SWL group) were analyzed for HRQoL. SF-36 questionnaire was applied pre-operatively, post-operative day 1 (POD1) and 1 month after the procedure. At POD1, RIRS group was associated with lower scores of role functioning/physical (p=0.008), role functioning/emotional (p=0.047) energy/fatigue (p=0.011), social functioning (p=0.003) and pain (p=0.003) when compared to the SWL group. At post-operative 1 month, only pain and emotional well-being scores (p=0.012 and p=0.011, respectively) in the RIRS group were lower than SWL group. Up to one month after procedure, HRQoL scores were favorable for SWL compared to RIRS.

This study showed that both treatments had a negative impact on HRQoL of the patients in a short term and that patients undergone RIRS scored lower that patients undergone SWL. However, this study used a non-specific questionnaire and only analyzed successfully treated patients. It would be interesting to know if a specific questionnaire for urinary stone patients would produce the same results. In order to help us decide which treatment would be more suitable, it would be important to consider HRQoL outcomes also in the patients with unsuccessful treatment. It is time to include HRQoL evaluation in RCT of interventional stone treatment.
